# Readmissions due to hyperemesis gravidarum: a nation-wide Finnish register study

**DOI:** 10.1007/s00404-022-06448-w

**Published:** 2022-02-28

**Authors:** Miina Nurmi, Päivi Rautava, Mika Gissler, Tero Vahlberg, Päivi Polo-Kantola

**Affiliations:** 1 Department of Obstetrics and Gynecology, Turku University Hospital and University of Turku, Turku, Finland; 2 Centre for Population Health Research, University of Turku and Turku University Hospital, Turku, Finland; 3 Turku Clinical Research Centre, Turku University Hospital, Turku, Finland; 4 THL Finnish Institute for Health and Welfare, Helsinki, Finland; 5 Division of Family Medicine, Department of Neurobiology, Care Sciences and Society, Karolinska Institute, Stockholm, Sweden; 6 Department of Clinical Medicine, Biostatistics, University of Turku and Turku University Hospital, Turku, Finland; 7 Department of Clinical Medicine/Public Health, University of Turku, Kiinamyllynkatu 10, 20014 Turku, Finland

**Keywords:** Hyperemesis gravidarum, Pregnancy, Miscarriage, Pregnancy termination, Ectopic pregnancy, Gestational trophoblastic disease

## Abstract

**Purpose:**

To evaluate the burden of illness caused by hyperemesis gravidarum (HG) and association of readmissions due to HG with maternal, environmental and pregnancy-related factors, and different pregnancy outcomes.

**Methods:**

Data of women with HG diagnosis in Finland, 2005–2017, were retrieved from health-care registers. Associations between readmissions due to HG and age, gravidity, parity, pre-pregnancy body mass index (BMI), smoking, marital status, socioeconomic status, municipality population, assisted reproductive technology (ART), and number and sex of fetuses were analyzed in pregnancies resulting in delivery. Admissions and readmissions due to HG in deliveries, gestational trophoblastic disease, ectopic pregnancies, miscarriages and pregnancy terminations were calculated.

**Results:**

10,381 pregnancies with HG diagnosis were identified: 9518 live births, 31 stillbirths, 8 cases of gestational trophoblastic disease, 16 ectopic pregnancies, 299 miscarriages, and 509 pregnancy terminations. Both outpatients and inpatients were included. Readmission occurred in 60% of pregnancies, inpatient readmission in 17%. Parity of ≥ 5, multiple gestation and female sex of fetus were associated with higher odds of readmission, maternal age 36–40 years, BMI ≥ 35 kg/m^2^, smoking and ART with lower odds of readmission. Of the 9549 pregnancies resulting in delivery, 33% involved at least one outpatient visit or inpatient episode after the first trimester, and 8% in the third trimester.

**Conclusion:**

The majority of women suffering from HG needed repeated medical care, often persisting after the first trimester. Our results provide practical information allowing clinicians to prepare for symptom duration beyond the first trimester and emphasize the importance of planning for eventual long-term treatment.

## Introduction

Hyperemesis gravidarum (HG) is the most common cause of hospitalization in the first trimester of pregnancy in pregnancies resulting in delivery [1], and sometimes continues even until birth [2–4]. Universally accepted definition of HG remains to be formulated, and an international collaborative group is working on a consensus definition. The first version was presented in the International Colloquium on Hyperemesis Gravidarum in 2019 and contained the following criteria: pregnant woman; other causes of nausea and vomiting were excluded; beginning of symptoms in early pregnancy; symptoms: nausea and vomiting (at least one of these severe); inability to eat/drink normally; strong effect on daily activity; signs of dehydration. Register studies, the present included, rely on clinical diagnoses as defined in the health-care system, which may differ according to local practices. Commonly cited definitions of HG include symptoms of nausea and vomiting, weight loss and dehydration [5–7]. In Finland, HG is diagnosed according to the ICD-10 classification of diseases, which titles the HG-related O21 diagnoses as “excessive vomiting in pregnancy” [8, 9]. HG is a relatively rare condition, estimated to occur in 0.3–3.6% of pregnancies [10, 11]. In Finland, HG hospitalization rate of 0.7% [12] and overall incidence of 1.3% have been reported [11]. Readmission rates from 13 to 34% have been observed [4, 7, 13–15], and average length of hospitalization has been reported to be 2–5 days [1, 7, 13, 16, 17]. Curative treatment for HG has not been discovered, and current treatment strategies aim at relieving symptoms and alleviating complications of HG, such as dehydration and malnutrition [18–20]. There are currently no official guidelines for diagnosis or treatment of HG in Finland, but hospitalization criteria and protocols regarding antiemetic medication, as well as hydration and nutrition have recently been described [21]. As women suffering from HG are keenly looking forward to their symptoms resolving, they would welcome a realistic estimation of how long the need of medical care is likely to continue. However, knowledge about symptom duration and risk factors for readmission due to HG is sparse. Young maternal age, low socioeconomic status, Asian or Black ethnicity, female fetus and multiple pregnancy have been found to be associated with higher risk of readmission [4]. In some studies, nulliparous women had higher readmission risk [4, 22], but in others, neither age nor parity were associated with readmission risk [7, 13]. In one study, rehospitalization risk increased if the first hospitalization occurred before 9 weeks’ gestation, was longer than 2 days, and if the woman had had a previous HG pregnancy [23].

Data about pregnancies not resulting in live birth in association with HG are limited as well. In one study, stillbirth was more common among HG patients [17], but in another, no association was found [24], and in one large study, women with HG had lower risk of stillbirth [25]. Symptoms of HG occur in gestational trophoblastic disease [26–28], whereas in ectopic pregnancy they are not typical [29, 30]. Nausea and vomiting appear to be associated with lower risk of miscarriage [31–34]. In some cases, HG has led to pregnancy termination [15, 35].

Our objectives were to estimate the number and duration of admissions and readmissions due to HG, to assess factors associated with readmissions, and to evaluate the associations between pregnancy outcomes and readmissions. These objectives all aimed at helping physicians to advise and treat HG patients concerning the course of HG symptoms and to understand the burden of illness due to HG in medical care.

## Materials and methods

STROBE guidelines [36] were followed in research and reporting of this study. The study plan was evaluated and approved by the Ethical committee of Hospital District of Southwest Finland (43/180/2011). Our data sources were the Hospital Discharge Register, the Medical Birth Register and the Register of Induced Abortions, used with permission of the Finnish Institute for Health and Welfare (THL/658/5.05.00/2012; THL/372/5.05.00/2018) as in our earlier study [11]. Data linkage between registers was performed using each woman’s unique personal identity code which is given to all citizens and permanent residents at birth or immigration and included in all Finnish health-care registers.

**Fig. 1:**
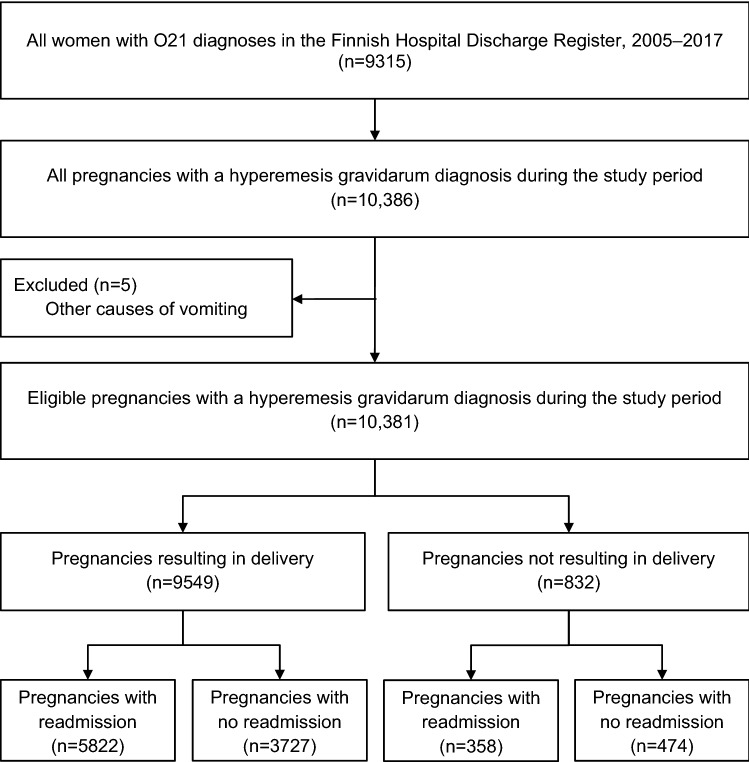
Flowchart of the study

All pregnancies (*N* = 10,381) with an HG discharge diagnosis (ICD-10 diagnosis codes used in Finland for clinical diagnosing of HG during the study period: O21, O21.0, O21.1, O21.2, O21.8 and O21.9) [8, 9] in the Hospital Discharge Register between years 2005 and 2017 were included in the study, regardless of the outcome. Both outpatient visits and inpatient episodes were included. The outcomes were determined by combining the HG diagnosis data with other register data: information about live births and stillbirths was drawn from the Medical Birth Register, pregnancy termination data from the Register of Induced Abortions, and diagnoses related to gestational trophoblastic disease, ectopic pregnancy and miscarriage were retrieved from the Hospital Discharge Register. Five pregnancies with another cause of vomiting than HG (four cases of gallstones and one case of pancreatitis) were excluded (Fig. 1).

The numbers of outpatient visits and inpatient episodes due to HG were calculated per 10,000 woman-years. Duration of inpatient episodes was calculated as days. An overnight inpatient episode was calculated as 2 days. The numbers of outpatient and inpatient episodes, and the total number of days spent in a hospital, were calculated per pregnancy. To account for the duration of pregnancy, the number of admissions were calculated per total number of pregnancy weeks in those pregnancies for which the information was available, i.e., pregnancies resulting in delivery and pregnancy terminations, as gestational week is not recorded in the Hospital Discharge Register in miscarriages, and duration of the condition in gestational trophoblastic disease or ectopic pregnancy is not recorded. Trimester-specific numbers of outpatient visits and inpatient episodes were calculated in pregnancies resulting in delivery and pregnancy terminations. Readmission rates in stillbirths, gestational trophoblastic disease, ectopic pregnancies, miscarriages and pregnancy terminations were compared to the readmission rate in live births.

In pregnancies for which the data were available, i.e., pregnancies resulting in delivery, the associations between readmissions and maternal, environmental and pregnancy-related factors were evaluated: maternal age in years (≤ 20; 21–25; 26–30; 31–35; 36–40 and ≥ 41), gravidity (number of pregnancies, including the present), parity (number of deliveries, including the present), pre-pregnancy body mass index (BMI) (< 18.5 kg/m^2^; 18.5–24.9 kg/m^2^; 25–29.9 kg/m^2^; 30–34.9 kg/m^2^ and ≥ 35 kg/m^2^), smoking (no; yes, but quit during the first trimester; yes, continued smoking after the first trimester), marital status (living/not living with partner), socioeconomic status based on standard classification of maternal occupation by Statistics Finland (upper-level white-collar workers, e.g., specialists and management level; lower-level white-collar workers, e.g., office staff; blue-collar workers, e.g., manual laborers; at home; other), municipality population (< 10,000 inhabitants; 10,000–99,999 inhabitants; ≥ 100,000 inhabitants), assisted reproductive technology (ART) (no/yes), number of fetuses (one; two or more) and sex of fetuses in singleton pregnancies (one male; one female) and multiple pregnancies (all male; all female; both sexes). Pregnancies involving only one outpatient visit or inpatient episode (no readmission) were compared to pregnancies involving more than one outpatient visit or inpatient episode (Table 1).

**Table 1:** Readmissions due to hyperemesis gravidarum according to pregnancy outcomes

Outcome	HG pregnancies, total	HG pregnancies, readmission^a^	Readmission rate, %	OR (95% CI) Compared to live birth
Live birth	9518	5803	61	Ref
Stillbirth^b^	31	19	61	1.02 (0.50–2.09)
Gestational trophoblastic disease	8	3	38	0.45 (0.13–1.60)
Ectopic pregnancy	16	4	25	0.21 (0.07–0.64)
Spontaneous abortion	299	121	40	0.44 (0.34–0.55)
Pregnancy termination	509	230	45	0.51 (0.43–0.61)
Total	10,381	6180	60	

*HG* hyperemesis gravidarum, *OR* odds ratio, *CI* confidence interval, *Ref* reference

^a^Readmission: more than one outpatient visit or hospitalization due to hyperemesis gravidarum during one pregnancy. The effect of duration of pregnancy in those pregnancies for which the information was available, i.e., pregnancies resulting in delivery and pregnancy terminations, is presented in Fig. 5

^b^Stillbirth: includes pregnancies with at least one stillborn fetus: 26 singleton stillbirths, four pregnancies with one live and one stillborn fetus, and one pregnancy with two live and one stillborn fetus

The associations of maternal, environmental and pregnancy-related factors in pregnancies resulting in delivery with readmission were analyzed using univariable and multivariable binary logistic regression: factors with a *p* value < 0.10 in the univariable analysis were included in the multivariable model. Socioeconomic status was excluded from the multivariable model due to large amount of missing data. Otherwise, missing data were rare and not imputed. Pregnancy outcome comparisons were analyzed with binary logistic regression. Generalized estimating equations were used to account for the repeated pregnancies of the women. Results are presented using odds ratios (OR) with 95% confidence intervals (CI). The change in the number of outpatient visits/10,000 woman-years and inpatient episodes/10,000 woman-years during the study period was tested using Poisson regression. Statistical analysis was performed with SAS System for Windows, version 9.4 (SAS Institute Inc., Cary, NC).

## Results

**Fig. 2:**
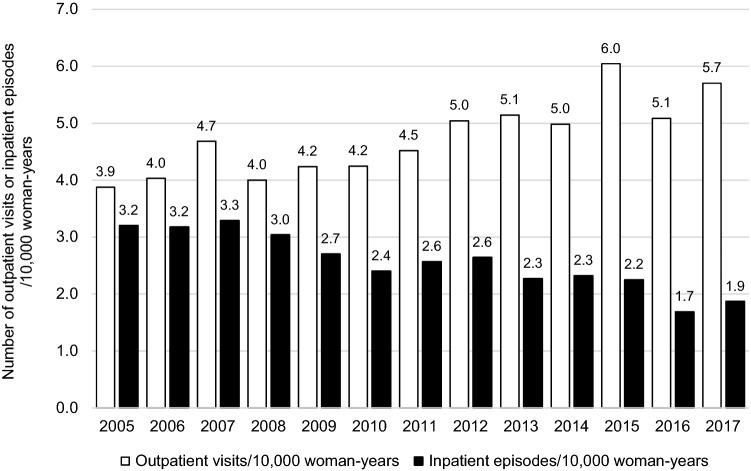
Outpatient visits and inpatient episodes due to hyperemesis gravidarum in Finland, 2005–2017

During the study period, there were altogether 16,853 outpatient visits (on average 1296 outpatient visits/year, equaling 4.7 outpatient visits/year/10,000 woman-years) and 9101 inpatient episodes (on average 700 inpatient episodes/year, equaling 2.6 inpatient episodes/10,000 woman-years) due to HG (Fig. 2). Altogether, 10,381 pregnancies involved at least one outpatient visit or inpatient episode. Of them, 9549 pregnancies resulted in delivery (9518 live births and 31 stillbirths). In pregnancies resulting in delivery in Finland, the incidence of HG was 1.3% and the recurrence rate of HG was 22%, as previously reported [11]. Of pregnancies diagnosed with HG, 832 did not result in delivery, and of these, 8 cases were gestational trophoblastic disease, 16 ectopic pregnancies, 299 miscarriages, and 509 pregnancy terminations.

Out of the 10,381 pregnancies diagnosed with HG, there were more than one outpatient visit and/or inpatient episode in 6180 pregnancies (60%). Inpatient readmission occurred in 1728 pregnancies (17%). Readmissions were most common in pregnancies resulting in live birth (61%) or stillbirth (61%) and lowest in ectopic pregnancies (25%) (Table 1).

**Fig. 3:**
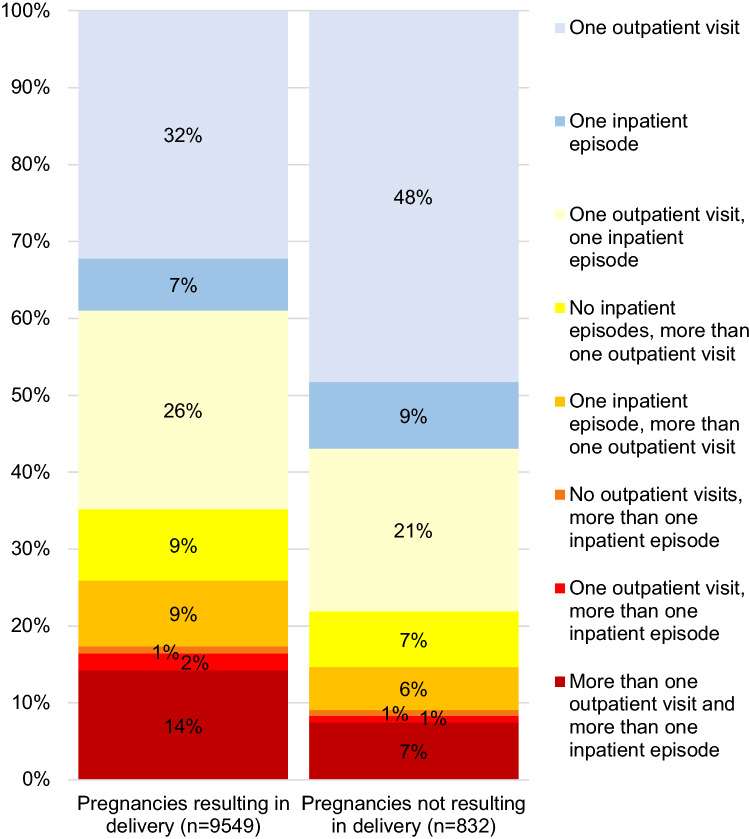
Admissions and readmissions due to hyperemesis gravidarum in 2005–2017

Frequencies of outpatient care and inpatient episodes are presented in Fig. 2. An increase in outpatient visits and decrease in inpatient episodes were observed: from 2005 to 2017, the number of outpatient visits increased from 3.9/10,000 woman-years to 5.7/10,000 woman-years (*p* < 0.0001), whereas the number of inpatient episodes decreased from 3.2/10,000 woman-years to 1.9/10,000 woman-years (*p* < 0.0001) (Fig. 2). Of the pregnancies resulting in delivery, 41% involved only outpatient visits, 51% involved both outpatient visits and inpatient episodes, and 8% only inpatient episodes. Of the pregnancies not resulting in delivery, 56% involved only outpatient visits, 35% involved both outpatient and inpatient care, and 9% only inpatient episodes (Fig. 3). The median number of outpatient visits or inpatient episodes was two per pregnancy, range 1–35. Separately, the median number of outpatient visits per pregnancy was 1, range 0–32, and the median number of inpatient episodes per pregnancy was 1, range 0–17. The median length of inpatient episodes was 3 days, range 1–129 days.

**Fig. 4:**
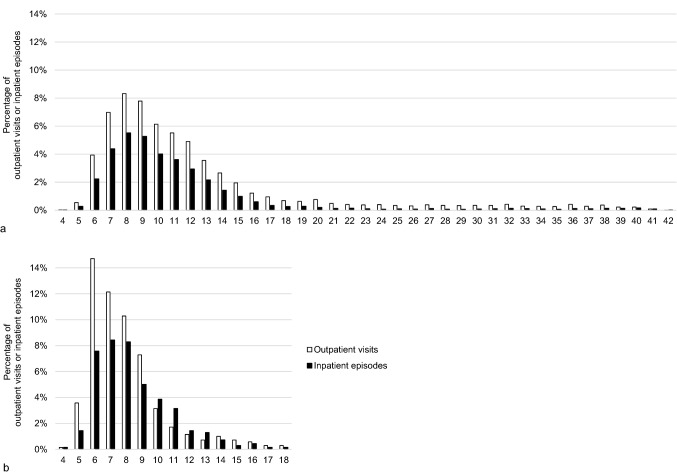
Timing of outpatient and inpatient care due to hyperemesis gravidarum per pregnancy week in pregnancies resulting in delivery (**a**) and in terminated pregnancies (**b**)

**Fig. 5:**
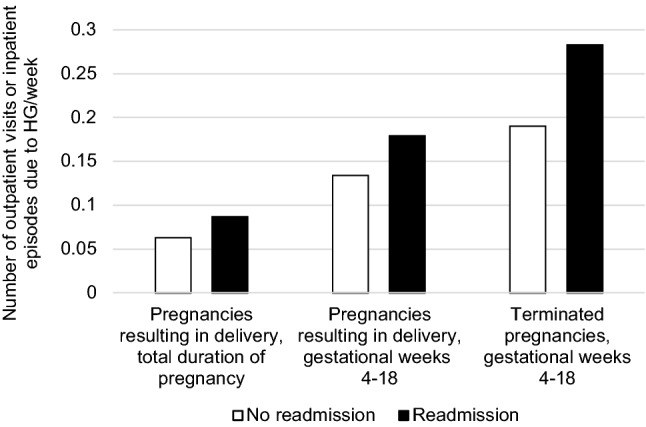
Number of hyperemesis gravidarum diagnoses per pregnancy week in pregnancies resulting in delivery and terminated pregnancies

The majority of outpatient visits and inpatient episodes occurred in the first trimester (Fig. 4). Of the 9549 pregnancies resulting in delivery, HG diagnosis was recorded only in the first trimester in 6351 pregnancies (67%), in the first and second trimesters in 921 pregnancies (10%) and from the first to third trimester in 262 pregnancies (3%); 1514 pregnancies (16%) had an HG diagnosis only in the second trimester, 389 pregnancies (4%) only in the third trimester and 112 pregnancies (1%) in the second and third but not in the first trimester. Most of the pregnancy terminations, 427 of 509, took place in the first trimester. Of the 82 pregnancies terminated in the second trimester, HG diagnosis was recorded only in the first trimester in 56 pregnancies (68%), in the first and second trimesters in 5 pregnancies (6%) and only in the second trimester in 21 pregnancies (26%). The number of admissions per gestational week was higher in terminated pregnancies compared to pregnancies resulting in delivery (Fig. 5).

In multivariable analysis of maternal, environmental and pregnancy-related factors in pregnancies resulting in delivery, parity of five or more, multiple gestation and female sex of the fetus were associated with higher odds of readmission, whereas maternal age of 36–40 years, pre-pregnancy BMI of 35 or more, smoking during pregnancy and ART were associated with lower odds of readmission (Table 2). Compared to upper-level white-collar, white-collar and blue-collar workers had slightly lower odds of readmission in the univariable analysis (Table 2), but due to high number of missing data, the variable was excluded from the multivariable model. Gravidity, marital status and municipality population did not show any association with readmission (Table 2).

**Table 2:** Results of univariable and multivariable analysis: associations between readmission due to HG and maternal, environmental and pregnancy-related factors in pregnancies resulting in delivery

Characteristic	Readmission^a^, all health-care services		OR, univariable	OR, multivariable^b^
	Yes (*N* = 5822)	No (*N* = 3727)		
	*N* (%)	*N* (%)	(95% CI)	(95% CI)
Age, years				
≤ 20	337 (5.8)	231 (6.2)	0.90 (0.75–1.08)	1.04 (0.85–1.27)
21–25	1321 (22.7)	907 (24.3)	Ref	Ref
26–30	1975 (33.9)	1201 (32.2)	0.88 (0.79–0.99)	0.92 (0.82–1.04)
31–35	1526 (26.2)	923 (24.8)	1.01 (0.91–1.12)	0.97 (0.87–1.09)
36–40	563 (9.7)	397 (10.7)	0.87 (0.75–1.01)	0.81 (0.70–0.95)
≥ 41	100 (1.7)	68 (1.8)	0.90 (0.65–1.23)	0.86 (0.60–1.21)
Gravidity (number of pregnancies, current pregnancy included)				
1	1870 (32.1)	1265 (33.9)	Ref	
2	1686 (29.0)	1043 (28.0)	1.07 (0.97–1.19)	
3	1011 (17.4)	658 (17.7)	1.02 (0.91–1.15)	
4	567 (9.7)	358 (9.6)	1.04 (0.90–1.21)	
≥ 5	687 (11.8)	403 (10.8)	1.13 (0.98–1.30)	
Unknown	1			
Parity (number of pregnancies resulting in delivery, current pregnancy included)				
1	2545 (43.7)	1720 (46.2)	Ref	Ref
2	1864 (32.0)	1183 (31.8)	1.04 (0.95–1.14)	1.03 (0.93–1.14)
3	851 (14.6)	516 (13.9)	1.09 (0.96–1.23)	1.12 (0.98–1.28)
4	292 (5.0)	181 (4.9)	1.05 (0.87–1.27)	1.14 (0.93–1.40)
≥ 5	268 (4.6)	126 (3.4)	1.39 (1.–1.72)	1.41 (1.11–1.78)
Unknown	2	1		
Pre-pregnancy BMI, kg/m^2^				
< 18.5	266 (4.7)	166 (4.6)	1.02 (0.83–1.25)	1.05 (0.85–1.29)
18.5–24.9	3286 (58.3)	2077 (57.7)	Ref	Ref
25–29.9	1277 (22.7)	799 (22.2)	1.01 (0.91–1.12)	1.01 (0.91–1.13)
30–34.9	552 (9.8)	353 (9.8)	0.98 (0.85–1.13)	1.01 (0.87–1.17)
≥ 35 or more	254 (4.5)	208 (5.8)	0.77 (0.63–0.93)	0.77 (0.63–0.93)
Unknown	187	124		
Smoking during pregnancy				
No	5213 (91.7)	3137 (86.6)	Ref	Ref
Yes, but quit in first trimester	236 (4.1)	212 (5.9)	0.67 (0.56–0.82)	0.67 (0.55–0.81)
Yes, continued after first trimester	238 (4.2)	273 (7.5)	0.53 (0.45–0.64)	0.54 (0.44–0.65)
Unknown	135	105		
Marital status				
Living with partner	5220 (93.5)	3296 (94.1)	Ref	
Not living with partner	361 (6.5)	205 (5.9)	1.12 (0.94–1.34)	
Unknown	241	226		
Socioeconomic status				
Upper-level white collar	634 (17.4)	325 (15.4)	Ref	
White collar	1450 (39.8)	927 (43.9)	0.80 (0.68–0.94)	
Blue collar	518 (14.2)	334 (15.8)	0.80 (0.66–0.97)	
At home^c^	287 (7.9)	135 (6.4)	1.08 (0.85–1.38)	
Other^c^	751 (20.6)	392 (18.6)	0.98 (0.82–1.18)	
Unknown	2182	1614		
Municipality population				
< 10,000 inhabitants	799 (13.7)	534 (14.4)	Ref	
10,000–99,999 inhabitants	2471 (42.6)	1591 (42.9)	1.03 (0.91–1.17)	
≥ 100 000 inhabitants	2536 (43.7)	1582 (42.7)	1.05 (0.93–1.20)	
Unknown	16	20		
Assisted reproductive technology (ART)^d^				
No	5640 (96.9)	3583 (96.1)	Ref	Ref
Yes	182 (3.1)	144 (3.9)	0.81 (0.65–1.02)	0.77 (0.61–0.97)
Number of fetuses				
One fetus	5612 (96.4)	3632 (97.4)	Ref	Ref
Two or three fetuses	210 (3.6)	95 (2.6)	1.46 (1.14–1.86)	1.64 (1.27–2.12)
Sex of fetus, all pregnancies^e^				
Male	2612 (44.9)	1776 (47.6)	Ref	Ref
Female	3210 (55.1)	1951 (52.4)	1.12 (1.03–1.21)	1.13 (1.04–1.23)
Sex of fetus, singleton pregnancies^f^				
One fetus, male	2531 (45.1)	1735 (47.8)	Ref	
One fetus, female	3081 (54.9)	1897 (52.2)	1.12 (1.03–1.21)	
Sex of fetuses, multiple gestation^f^				
All male	45 (21.4)	26 (27.4)	Ref	
All female	86 (41.0)	36 (37.9)	1.31 (0.71–2.44)	
Both sexes	79 (37.6)	33 (34.7)	1.32 (0.70–2.48)	

*OR* odds ratio, *CI* confidence interval, *Ref* reference

^a^Readmission: more than one outpatient visit or inpatient episode due to HG during one pregnancy

^b^Age, parity, BMI, smoking, ART, number of fetuses and sex of the fetus were included in the multivariable model

^c^Socioeconomic status, at home: unemployed, retired, stay-at-home mother; other: including, e.g., entrepreneurs, farmers and students, for which socioeconomic status cannot be determined

^d^ART: yes = insemination, follicle stimulation or embryo transfer, or a combination of these

^e^In twin pregnancies, the sex of the firstborn fetus was included in the analysis

^f^Additional analyses, not included in the multivariable model due to collinearity with the sex and number of fetuses

## Discussion

The burden of illness due to HG was substantial: in the scale of Finland, with a population of 5.5 million inhabitants and 50,000–61,000 births/year in 2005–2017, more than a thousand outpatient visits and several hundred inpatient episodes each year present a considerable load for health services and strain for the pregnant women. In one-third of pregnancies, the need of care due to HG continued after the first trimester, and in nearly one-tenth of pregnancies in the third trimester, highlighting the value of preparing for long-term treatment of HG.

In line with previous studies, outpatient visits were more common than inpatient episodes [37, 38]. The inpatient readmission rate, 17%, is likely to correspond to more severe HG symptoms, as outpatient visits occurring after inpatient episodes may also include follow-up visits with diminished HG symptoms. However, as the total number of outpatient visits and inpatient episodes remained at the same level throughout the study, the observed increase of outpatient care and decrease of inpatient care may reflect current objectives of early and accessible treatment in health-care units near the patients rather than changes in overall severity of HG symptoms during the study period [38–40].

In our study, repeated care was needed in 60% of HG pregnancies. Earlier studies have reported lower readmission rates: 13% (14 of 109 women) [15], 20% (38 of 192 women) [13], 28% (34,704 of 121 885 women) [4], 32% (62 of 191 women) [7] and 34% (38 of 113 women) [14]. The differences are likely to result from methodological diversity: in most studies, only inpatient hospitalizations [4, 7, 13, 15] or emergency department visits [14] were analyzed, and the sources of data varied: single hospital [13–15], several hospitals [7] or nation-wide hospitalization data [4]. Our inpatient readmission rate, 17%, fell in the lower end of the readmission rates observed earlier [4, 7, 13–15], highlighting the effect of different inclusion and exclusion criteria between studies.

Young maternal age has been associated with higher risk of readmission due to HG [4], in line with our observation of a lower risk in the older age groups, although the difference was statistically significant only in the age group of 36–40 years. Earlier studies have been inconsistent concerning the effect of parity [4, 41–43], and in our study, only parity of five or more was associated with higher risk of readmission. ARTs have been shown to be associated with risk of HG in general [42], and in our earlier study, we found the same result when comparing pregnancies diagnosed with HG to pregnancies not diagnosed with HG [11]. In the present study, the novel finding of inverse association of ART with readmissions can imply that the symptoms may not persist a long time in these cases. The small number of ART pregnancies did not permit distinguishing between different ART techniques, limiting our ability to interpret possible effect of different biological reasons for use of ARTs. Our results about smoking matched earlier results: smoking has been associated with lower risk of HG in general [41]. In a large study by Fiaschi et al. [4], low socioeconomic status, Asian or Black ethnicity, female fetus and multiple pregnancy were found to be risk factors for readmission, and our results confirmed these results regarding the number and sex of fetuses. The observed higher number of admissions per gestational week in terminated pregnancies compared to pregnancies resulting in delivery may reflect the strain of HG as one factor affecting the pregnancy termination decision, but as register data does not permit analyzing women’s motives in such decisions, these results should be interpreted with caution. Furthermore, in previous studies, nausea and vomiting in pregnancy and HG have been inversely associated with miscarriage [32, 33], in line with our observation of lower readmission rate due to HG in pregnancies resulting in miscarriage.

Although nausea and vomiting are often considered to resolve after the first trimester and studies tend to focus on the first trimester [14, 44], previous studies have shown that symptoms frequently persist past mid-pregnancy or even until birth [2–4]. In interview studies, women suffering from HG have expressed frustration when their symptoms continue longer than expected, highlighting the value of realistic evaluation of duration of HG [45, 46]. Our results provide helpful information regarding this question allowing clinicians to reflect the readmission risk and symptom duration in each woman’s individual situation. As many of the above-mentioned risk factors are not influenceable, these results cannot be employed for diminishing an individual woman’s readmission risk due to HG, but rather as tools for evaluating if her symptoms will be likely to persist and need repeated care.

The Finnish health-care registers’ high coverage and reliability are valuable assets for register-based studies [47]. We used nation-wide register data of outpatient visits and inpatient episodes of women admitted for HG, permitting admissions in different services to be merged. In Finland, primary health care is publicly funded and organized in health-care centers, including prenatal care units for pregnancy follow-up [48]. Virtually, all pregnant women attend routine pregnancy care organized by specialized maternity health-care nurses and midwives, with regular check-ups by physicians. Mild nausea and vomiting can be treated in primary care, and women with HG are referred to specialized obstetric clinics, led by specialists in obstetrics, where they can receive outpatient care or be admitted to a hospital. Registering a diagnosis following national guidelines is obligatory, and verified discharge diagnoses are systematically collected into centralized registers organized by the Finnish Institute of Health and Welfare [49]. Data linkage between registers enabled us to connect HG diagnoses to varied pregnancy outcomes. Analyzing both outpatient visits and inpatient episodes in the entire population allowed us to get a comprehensive picture of need of care due to HG, not limited to hospitalizations or certain service providers. The most important limitation to consider when assessing need of care using register data is the possible underestimation, as those women suffering from HG symptoms who did not contact health care or did not receive diagnosis were absent in the data. For some research questions, registers are not optimal. For instance, estimation of the number of miscarriages is limited by the extent to which they are recorded in health-care registers, and as not all miscarriages are clinically recognized [50], their number is likely to be underestimated. Also, the data structure did not permit adjustment for pre-existing or gestational comorbidities, symptom severity or treatment modalities, and assessing these questions requires a different study design. The small number of gestational trophoblastic disease and ectopic pregnancies diagnosed with HG suggest that results regarding these outcomes are to be interpreted with caution.

Definition of HG is essential for comparability of results, and different diagnostic and treatment practices may limit generalizability of findings. In Finland, HG is diagnosed according to the 10th version of the International Statistical Classification of Diseases and Related Health Problems (ICD). Different inclusion and exclusion criteria in HG studies have been common due to lack of consensus about the definition [51, 52]. The first consensus definition of HG presented in the Third International Colloquium on Hyperemesis Gravidarum is primarily intended for prospective clinical studies and cannot be retrospectively implemented in register research. There are currently no official hospitalization criteria or clinical guidelines for diagnosis or treatment of HG in Finland, but current practices and recommendations have been described in the primary Finnish Medical Journal Duodecim [21]. Women with HG can receive treatment, such as intravenous hydration or medication, in both outpatient and hospital settings, and the dichotomy turned out to be somewhat artificial: our analysis showed that in Finland, the majority of women with HG were treated in both settings.

In conclusion, our results reveal that readmissions due to HG are common and the need of medical care often continues after the first trimester of pregnancy. These findings emphasize the importance of planning for eventual long-term treatment of HG.

## Data Availability

Adhering to the EU General Data Protection Regulation (GDPR) and Finnish legislation concerning sensitive data such as health-related information, the authors are not authorized to share the data. The Finnish Institute for Health and Welfare (THL) can, on a case-by-case basis, grant permission to use the registers for purposes of scientific research.
